# In-silico models of stem cell and developmental systems

**DOI:** 10.1186/1742-4682-11-1

**Published:** 2014-01-08

**Authors:** Yaki Setty

**Affiliations:** 1 Computational Systems Biology, Max-Planck-Institut für Informatik, Saarbrücken 66123, Germany

## Abstract

Understanding how developmental systems evolve over time is a key question in stem cell and developmental biology research. However, due to hurdles of existing experimental techniques, our understanding of these systems as a whole remains partial and coarse. In recent years, we have been constructing in-silico models that synthesize experimental knowledge using software engineering tools. Our approach integrates known isolated mechanisms with simplified assumptions where the knowledge is limited. This has proven to be a powerful, yet underutilized, tool to analyze the developmental process. The models provide a means to study development in-silico by altering the model’s specifications, and thereby predict unforeseen phenomena to guide future experimental trials. To date, three organs from diverse evolutionary organisms have been modeled: the mouse pancreas, the C. elegans gonad, and partial rodent brain development. Analysis and execution of the models recapitulated the development of the organs, anticipated known experimental results and gave rise to novel testable predictions. Some of these results had already been validated experimentally. In this paper, I review our efforts in realistic in-silico modeling of stem cell research and developmental biology and discuss achievements and challenges. I envision that in the future, in-silico models as presented in this paper would become a common and useful technique for research in developmental biology and related research fields, particularly regenerative medicine, tissue engineering and cancer therapeutics.

## Review

### Challenges in stem cell and developmental biology research

Developmental systems consist of a cell population that maintains the proper development, structure and function of multicellular organisms [1-3]. In these systems, cells act as the basic elements [4,5] and largely control development via two key mechanisms. The first is proliferation by which a single cell gives rise to two daughter cells. The second is differentiation by which a cell adopts a more specialized cell type [3]. These mechanisms are not independent; a proliferative cell may block differentiation and conversely, a cell at a specific differentiation stage may block proliferation. Moreover, the interplay between the two mechanisms varies between different systems and organisms [6-11].

**Figure 1:**
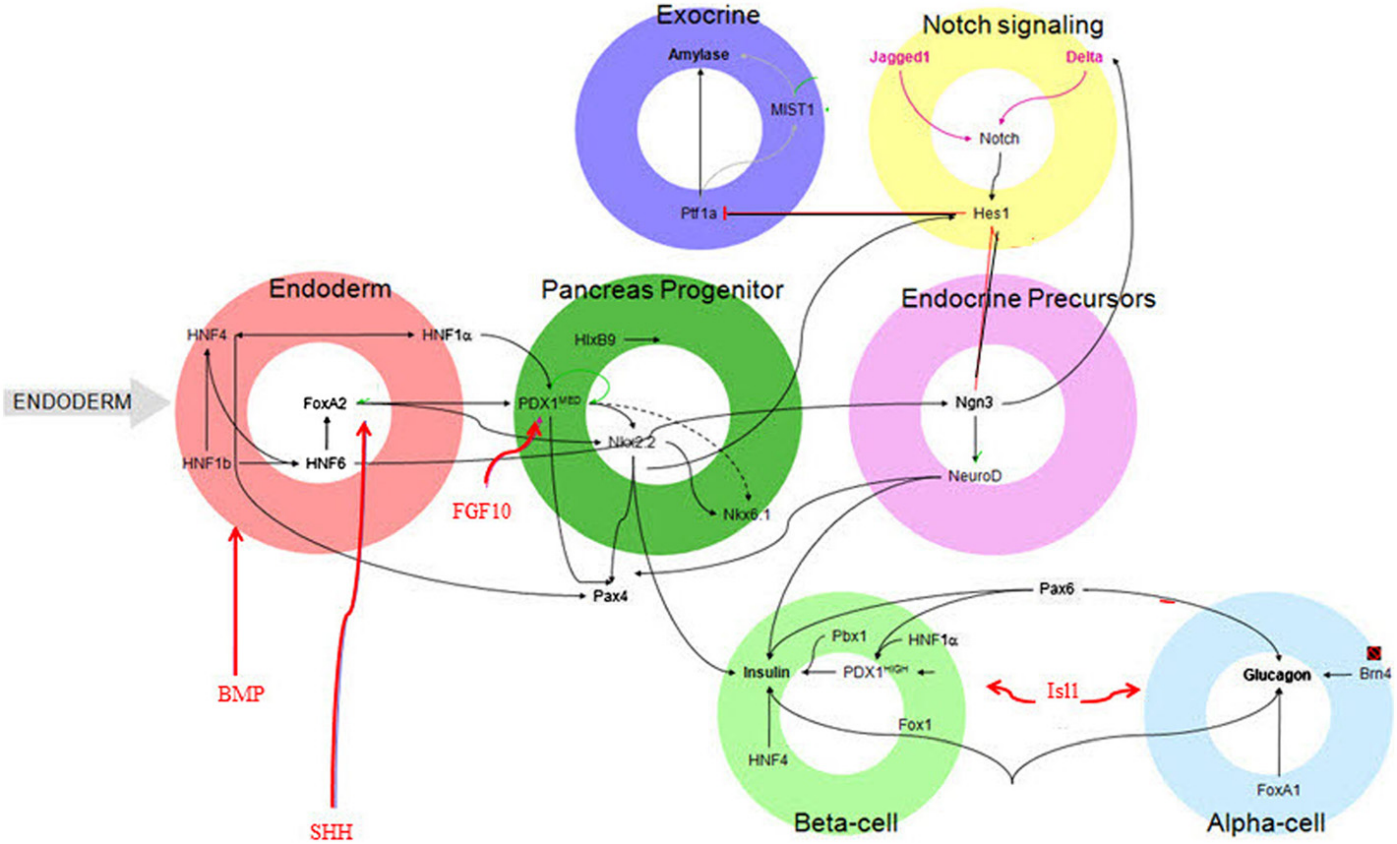
**An example of the genetic regulatory network in pancreatic development.** Interactions between cell-extrinsic cues (red arrows) and cell-intrinsic molecules (black arrows) mediate cellular differentiation. Endoderm cells (red) give rise to a pancreas progenitor cell (green) that enter the Notch signaling stage (yellow) to determine endocrine (purple) or exocrine (blue) fate. Endocrine cells then commit to a beta or alpha fate (reproduced from [6])).

The development of individual cells in the population is driven by the activity of molecular mechanisms, such as receptor activity, gene expression and protein degradation [2,12]. These mechanisms compose a complex network that implicates cell-extrinsic cues and cell-intrinsic pathways. Arduous experimental studies have identified key elements in the network and characterized molecular interactions (see e.g., a complex regulatory network that controls pancreatic development in Figure 1 (reproduced from [6])). Existing experimental techniques, however, are not yet fully able to overcome hurdles in generating data for multiple time points throughout the development. Most of the state-of-the-art time-point techniques are able to record merely a small number of cell divisions and longitudinal experimental studies that could generate time-dependent data are currently not yet feasible.

Therefore, the picture that emerges from the available experimental data is essentially static. The precise cellular mechanisms, their timing and how the regulate the system remain course [3]. Consequently, current knowledge cannot yet fully explain how the complex molecular network drives development over time. Key outstanding questions include: how do cells coordinate interactions over-time, how do molecular interactions drive structure formation and tissue patterning, and how does the interplay between signaling pathways affect the system.

In order to address these questions and to better understand the development process as a whole, we must assemble the existing knowledge into a complete system-level picture. This is the first step in exploring the molecular mechanisms that drive the cellular determination and how the system emerges from the collective activity of individual cells. The system-level picture highlights gaps in our understanding, and encourages the formation of hypotheses to bridge these gaps to direct experimental trails. In the long run, this research could help to understand the causes of improper control which may result in an abnormal development such as tissue degeneration and cancer [7-11].

### Related work

Interest has been increasing in the utilization of In-silico modeling applied to various biological systems [13]. Models were constructed for the electrical function of the heart [14], diffusion in the early Drosophila embryo [15], pattern formation in Zebrafish [16], dual perturbation experiments [17], and more. The challenges and advances in this approach for modeling were recently reviewed in several publications [13,18-20]. Most studies, however, study specific models that are built to describe a specific biological system or process, often cell-intrinsic ones [21-24]. A few have attempted a more generic approach. Among those are the Virtual Cell [25] and Cellerator [26] which utilize differential equations-based software environment for modeling cell biology.

Several related modeling efforts were aimed at understanding cell population dynamics. Gene- and protein-level mathematical models identified expression patterns in stem cell systems development [23,27]. A platform termed STORM was used to determine the number of epithelial stem cells in experimental systems [28]. Mathematical models are also used to infer patterns of stem cell divisions from clone size distributions [29,30] and stem cell development in the intestinal crypt [31-33]. Other in-silico models using various mathematical techniques simulated sub-systems involved in cell-regulation. Among these are models for cell motility and chemotaxis [34], the circadian rhythm [35], aspects in cell cycle mechanism [36] and cellular morphogenesis [37,38]. This work provides a platform to analyze and study the particular system in question and helps reveal insights.

However, due to the mathematical nature of many of these models, these in-silico models are often limited in their capability to capture dynamics in individual cells and rarely allow complex perturbations to predict spatial distributions of cells and complex phenotypes. Moreover, the majority of these modeling efforts consider the development only at single scale of the biological system and overlook effects of the related scale [39]. For example, modeling single cell behavior without accounting for the multiple interactions at the tissue scale. To realistically understand the entire development of cells in the tissue, there is a need for in-silico models that enable integration of the regulation of sub-systems into a comprehensive multi-scale dynamic model.

The in-silico models discussed here are different from the more traditional modeling efforts that often use toy-models to simplify complex process in order to seek hidden parameters or to test a specific hypothesis [18]. In particular, these models embody two key features: comprehensiveness and realism. The notion of comprehensive modeling guides us to construct models of the entire system rather than to focus on a specific aspect. The other notion, realism, forces us to verify that the model captures not only the overall behavior of the system, but also the behavior of the individual entities and their inter-relationships. These features generate simulations that can be analyzed in-silico by mimicking in-vivo experiments and predicting their outcome [13,14,39,40].

### In-silico models for stem cell and developmental systems

Tools that were originally designed for software engineering purposes provide a means to synthesize experimental information into in-silico models [41]. These models simulate the developmental process over time and space that emerges from the combined behaviors of the individually modeled entities [42]. Specifically, models of development and stem cell systems simulate how the tissue structure and function emerge from the collective activity in numerous individual cells [43].

The in-silico models reviewed here utilize software engineering tools to tackle dynamics and emergence in the process of development. The models simulate developmental systems by structuring known biological information in designated scale: (1) molecular mechanisms implicated in the regulatory network (2) proliferation, fate determination and motility, and (3) anatomic constraints and cell-extrinsic cues are defined in a grid that overlays the structure. Inter-scale and intra-scale interactions events that emanate from elements in one scale are translated to events at the other scales. Thus, the collective intra- and inter-scale events drive the simulation over time yielding a dynamic representation of the process (see [43] for a detailed discussion of this modeling approach).

To date, we have constructed in-silico models for three distinct organs from evolutionary diverse organisms: the mouse pancreas [44], the C. elegans gonad [45], and partial rodent brain development [46]. Each of the in-silico models recapitulated experimental observations and provided a means to investigate, factually and hypothetically, how molecular interactions over the regulatory network drive tissue patterning and structure formation. Notably, these aspects of development cannot be immediately drawn from the isolated parts, such as intrinsic molecular regulation or a single cell determination; indeed these aspects must be studied at the multicellular system-level. Our in-silico analyses anticipated experimental observations and revealed intriguing insights which provided testable predications. Notably, the same underlying modeling approach (described in [43]) was suitable to beneficially model the three subjects, though they are different in nature. This suggests that this is a unique modeling generic approach that may serve to model in-silico stem cell and developmental systems in various organisms. This review summarizes the models and briefly presents achievements of each.

### Modeling mouse pancreatic organogenesis in four dimensions: structure formation and tissue patterning

The pancreas evolves from a set of precursor cells into an intricate tissue with a unique structure and specialized function [6,47]. This process advances in multiple cells simultaneously through interactions in an intracellular complex molecular network that regulates proliferation and differentiation. In mice, pancreatic development initiates when cells at a designated area on the endodermal gut tube detect cell-extrinsic cues that trigger their specification process which determines their pancreatic fate. In parallel, the cells aggregate to form the tissue structure. Initially, cells commit for an endocrine or an exocrine fate and later adopt their final fate [6,48-52].

Our in-silico study of pancreatic organogenesis constructed an executable, visual and dynamic four-dimensional model (with time being the fourth dimension) [44, (Setty Y, Magenheim J, Harel D, Dor Y: In-silico study of pancreatic morphogenesis and differentiation. In preparation 2014)]. Our model covered the development of the dorsal pancreas from a flat endodermal tissue to the complex structure with specialized tissues organized in an intricate pattern. The modeling was informative and provided a framework to postulate hypotheses and test them in-silico.

**Figure 2:**
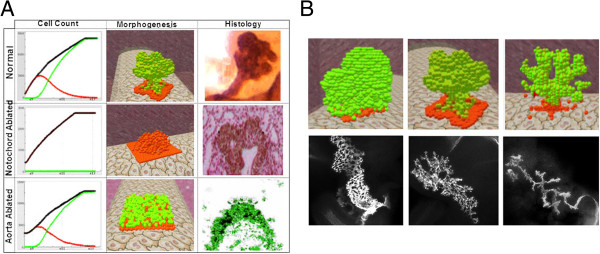
**Structural formation of pancreatic organogenesis, in-silico results vs. in-vivo (reproduced from [**[44]**]). A**. Pancreas organogenesis under normal conditions (top), pancreatic organogenesis under notochord (middle) and aorta ablation (bottom). Left column: red indicates undifferentiated cell count, green indicates pancreatic cell count and black indicates the total cell count. Middle column: red spheres designate an undifferentiated cell, green spheres designate a pancreatic cell. **B**. Relations between pancreatic organogenesis and blood vessels layout. Three distinct structures of pancreas in-silico prediction (top) vs. in-vivo data (bottom): highly lobulated structure under a reduced vascular network (left) a cauliflower-like shaped under normal conditions (middle) and highly branched structure under enhanced vascular network (right).

As part of the model analysis process, we compared the model’s output against experimental and theoretical observations. The comparison revealed a qualitative agreement with variety of experimental data from diverse sources. For example, the model anticipated the relations between the structural development and tissues in the extracellular matrix (Figure 2A). Simulation results for normal development generated a lobulated structure consisting of pancreatic cells (Figure 2A, top), whereas disabling the aorta or the notochord resulted with a distinct structure. When the impact of the notochord tissue was disabled, the pancreas initiated the structural formation process but terminated as a primitive bud that consisted of endoderm cells (Figure 2A, middle). In contrast, when the aorta was disabled, the endoderm cells triggered the differentiation process and committed to the pancreatic progenitor fate but the tissue remained flat (Figure 2A, bottom). Similar tissue patterning was observed in in-vivo experiments in which the notochord and the aorta were ablated (Figure 2A).

The output of the model and the modeling process pointed to several novel hypotheses and testable predictions. For instance, we hypothesized a possible role of the vascular network to explain pancreatic morphogenesis [44]. Using the model, we predicted the formation of distinct structures for altered layout of the vascular network. Specifically, we predicted that a condensed development of blood vessels leads to a massive lobed pancreas and that a branched blood vessel layout would give rise to a highly branched structure (Figure 2B). This hypothesis has recently gained increasing experimental evidence [53,54] which validates the impact of the vascular network on the pancreatic structure. In particular, a recent experimental study showed that the blood vessel network in the pancreatic mesenchyme restrains the pancreatic branching and development [55]. Similar to the model results, the experimental findings showed that mice whose vascular network is reduced developed a massive pancreatic tissue, whereas mice whose vascular network is enhanced developed a distinct branched structure (Figure 2B).

### Modeling C. elegans gonad development: population dynamics of a stem cell population

**Figure 3:**
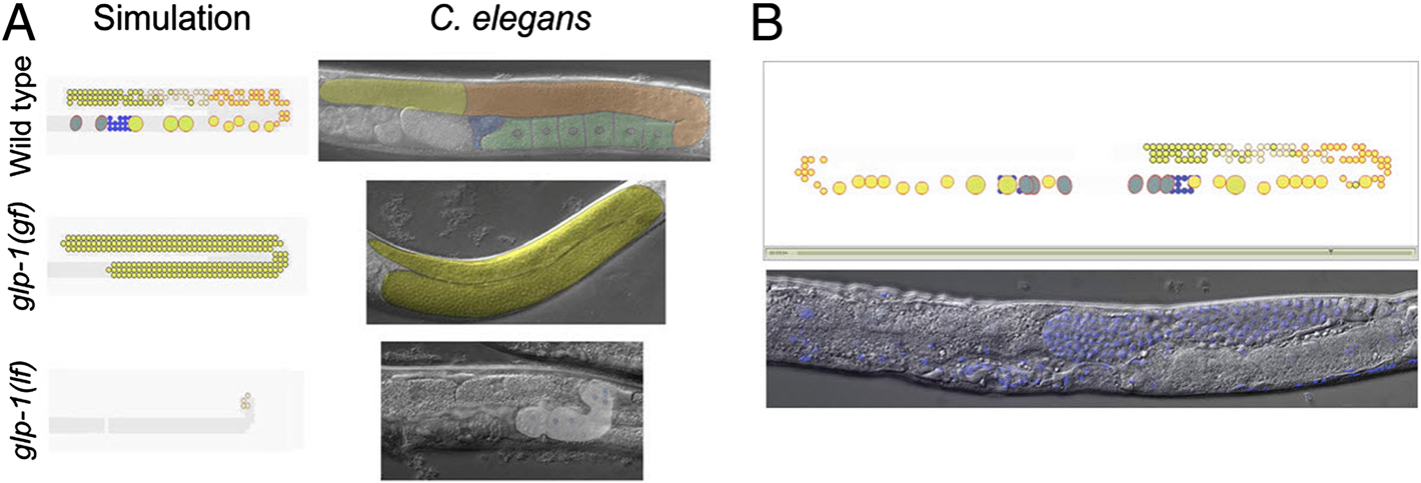
**Simulation of the C. elegans germline recapitulates developmental patterns (reproduced from [**[45]**]). A**. Simulation (left) and live adult hermaphrodites (right) under baseline/wild-type, elevated, and reduced GLP-1 receptor activity (top, middle, and bottom, respectively). Cell (left) and mask (right) colors indicate proliferative zone (yellow), meiotic prophase (orange), oocytes (yellow-green with orange outline), and sperm (blue). **B**. Examples of the proliferative zone retained in one arm and lost in the other in the simulation vs. C. elegans mutant. (Top) Gonad development as displayed by the simulation (Bottom) the lag2(q420) mutant which display similar phenotype.

C. elegans germline (that is, the lineage of cells that give rise to gametes) is a self-renewing cell population located in the gonad of the nematode [56-58]. The germline develops from a small number of cells that form two U-shaped arms. The germline stem cell system implicates proliferation of cells whereby cells divide and create new instances, and differentiation of germ cells from precursor cells to mature gametes. Cells that initiated the differentiation process go through three phases as they are ‘pushed’ proximally by the proliferation of neighboring cells toward the uterus at the central part of the structure. Precursor cells (Figure 3A, Top-Right; yellow mask) enter early meiosis stage, from which they adopt a pachytene fate (Figure 3A, Top-Right; orange mask). Finally, the cells mature as oocytes (Figure 3A, Top-Right; green mask) that eventually cross the spermetica (Figure 3A, Top-Right; blue mask) to enter the uterus.

**Figure 4:**
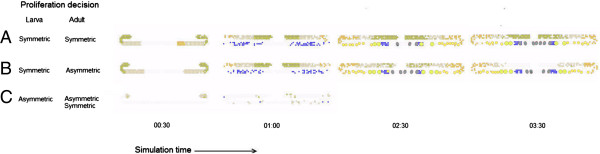
**Three distinct population dynamics under symmetric and asymmetric proliferation decision at the larva and adult stage. A**. Simulation under symmetric proliferation decision in which each division gives rise to an identical daughter proliferation and solely depends on receptor activity. The simulation displays a sustainable proliferative zone at the entire simulation time (tested in > = 30 simulations over time period of 10 min). **B**. Simulation under symmetric proliferation decision at larvae stages (simulation time < 1:30 min) and asymmetric proliferation decision in which the daughter cell immediately differentiates at the adult stage (simulation time > = 1:30 min). The simulation develops normally at the larvae stages and gradually lost the proliferative zone at the adult stage. **C.** Simulation under asymmetric proliferation decision at the larval stage. The simulation displays an aberrant development in which the cell population is lost early at the larvae stages and cannot maintain normal cell population. Under these conditions the simulation lasts 1:30 min in total (i.e., all cells were vanished from the gonad structure).

We built an in-silico model based on the known developmentally regulated aspects of morphology, signaling pathways, and proliferation behavior. The model captured the general developmental progression of germline development as a dynamic process over time [45] (Figure 3A, Top). We first altered the model design in ways that mimic known mutants. For instance, we enhanced the activity of the key receptor and found that simulation develops a pattern consisting of proliferative cells that have blocked differentiation (Figure 3A, Middle). In contrast, we reduced the activity of the receptor and found that the simulation develops only a few differentiated cells (Figure 3A, Bottom). These observations are consistent with mutant phenotypes whose receptor was engineered to gain and lose function (Figure 4A).

Model analysis provided possible explanations for laboratory observations, including complex temporal patterns of differentiation. For instance, under partial reduced activity of the key receptor, the model pointed to an unforeseen “all‒or‒none” phenotype. In this phenotype the cell population was sustainable in one arm of the gonad and was unsustainable in the other. In a subsequent in-vivo experiment, this phenotype was observed in a mutant whose receptor activity was engineered to partially reduce its function (Figure 3B). Base on the model output, we hypothesized that population dynamics within an individual gonad arm may contribute to an apparent “all‒or‒none” mutant phenotype. These unique dynamics are likely related to a critical minimum number of cells within an individual gonad arm at a specific time.

The model provided a platform to test hypothetic ‘what-if’ scenarios. One of these scenarios contrasted the symmetric versus asymmetric proliferation behavior in different developmental stages. A symmetric proliferation decision implies that the mother cell gives rise to two identical cells, whereas, an asymmetric proliferation decision implies that the mother cell give rise to one identical cell and one differentiated cell. The model, consistent with experimental findings, anticipated that cells proliferate symmetrically in all stages of development. The emergent pattern under this proliferative cellular behavior maintained the proliferative zone at the distal parts of the each gonad arm over time (Figure 4 Top; yellow). Nonetheless, the model allowed a study of emergent behavior under altered proliferative decisions, namely under asymmetric proliferation that was recorded in other developmental systems. Initially, we tested a scenario in which cells proliferate symmetrically at the early stages of the development but proliferate asymmetrically in the adult stage. In this scenario, the development was normal at the early stages, but the proliferative zone in the adult showed an intermingled pattern of proliferative cells (Figure 4, Middle). Eventually the proliferative zone was unsustainable (i.e., the gonad could not maintain its structure over time).

Next, we set the model to proliferate asymmetrically both at the early and adult stages of development. In this scenario, the gonad could not develop normally at the early stages. The few proliferative cells could not achieve a critical population size to maintain the population dynamics and structure in the adult. The emergent pattern displayed an aberrant pattern predominantly governed by differentiated cells. Moreover, modifying the decision at the adult stage to a symmetric proliferation decision gave rise to a similar pattern. This finding suggests that asymmetric proliferation at the early stages severely damaged the development and could not be restored at the adult stage by symmetric proliferation (Figure 4, bottom).

### Modeling neuronal migration in the cortex: dynamics at the single-cell level

Neuronal migration, the process by which newly born neurons find their path to the surface of the brain, is highly dynamic and complex. The process begins when neuroblasts divide from their progenitor glial cells and move along the glial fiber from the sites of origin towards the intermediate zone where they enter a multipolar stage. In this stage, the neuroblasts disassociate from the glial fiber and migrate independently through the zone [59,60]. When neurons re-associate with the glial fiber they adopt a bipolar migration stage to migrate to the cortical plate, where they accumulate beyond their predecessors, forming layers in an inside-out manner [59,60].

**Figure 5:**
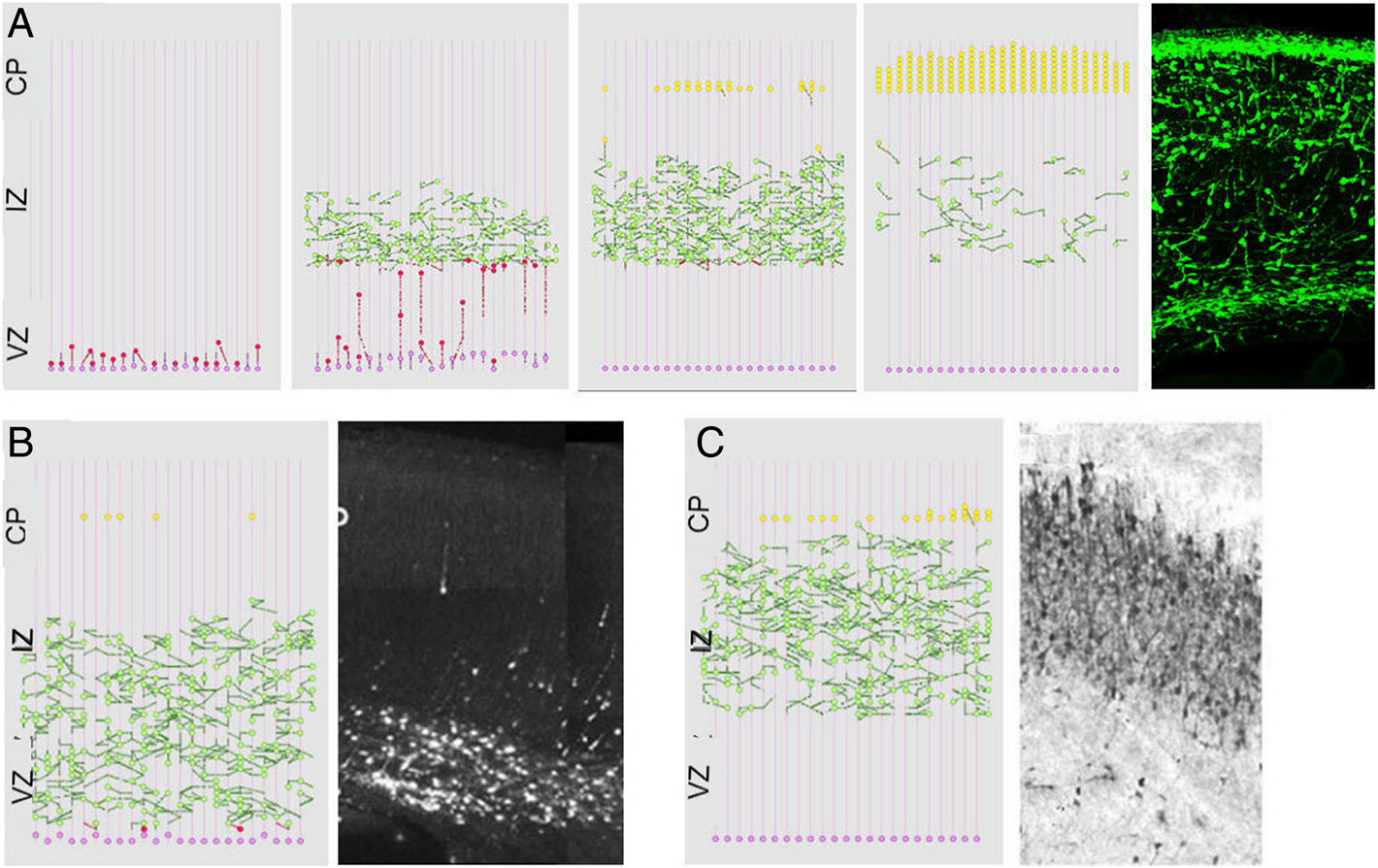
**Comparison of the in-silico model and experimental data of the neuronal migration process. A**. Four Snapshots of the neuronal migration simulation vs. histology of the embryonic mouse cortex. Radial progenitor glial cells (pink) proliferate asymmetrically giving rise to neuroblasts (red). Neuroblasts adopt a multipolar migration and move randomly stage (green). Multipolar neurons enter bipolar stage (yellow) and re-associate with the fiber, and accumulate at the pile surface. **B**. Lis1 mutants: (left) In-silico migration in Lis1-defective simulations vs. (right) histology of the cortex in Lis1 RNAi mutant mouse (reproduced with permission from [61]). **C**. In-silico migration under reduced DCX expression (left) vs. histology of the cortex in DCX RNAi mutant rats (right) (reproduced with permission from [62]).

In this project, we formulated a subset of the molecular factors based on their functionality in the process [46]. The model generated system-wide migration phases and cellular dynamics consistent with experimental observations (Figure 5, Top). Moreover, the simulation anticipated aberrant migration patterns under reduced activity of the two key regulators consistent with in-vivo experiments (Figure 5, Bottom). In particular, the model enabled us to analyze the system at the single neuron level. At this resolution, the model predicted that under reduced activity of the key regulator Lis1, newborn neuroblasts repeatedly associate and disassociate with the glial fiber before adopting a glial independent migration. The phenomenon varied between individual neurons. In the extreme cases, neurons immediately disassociate from the glial fiber or kept oscillatory association over the entire period. Intriguingly, this behavior was not explicitly programmed into the model.

**Figure 6:**
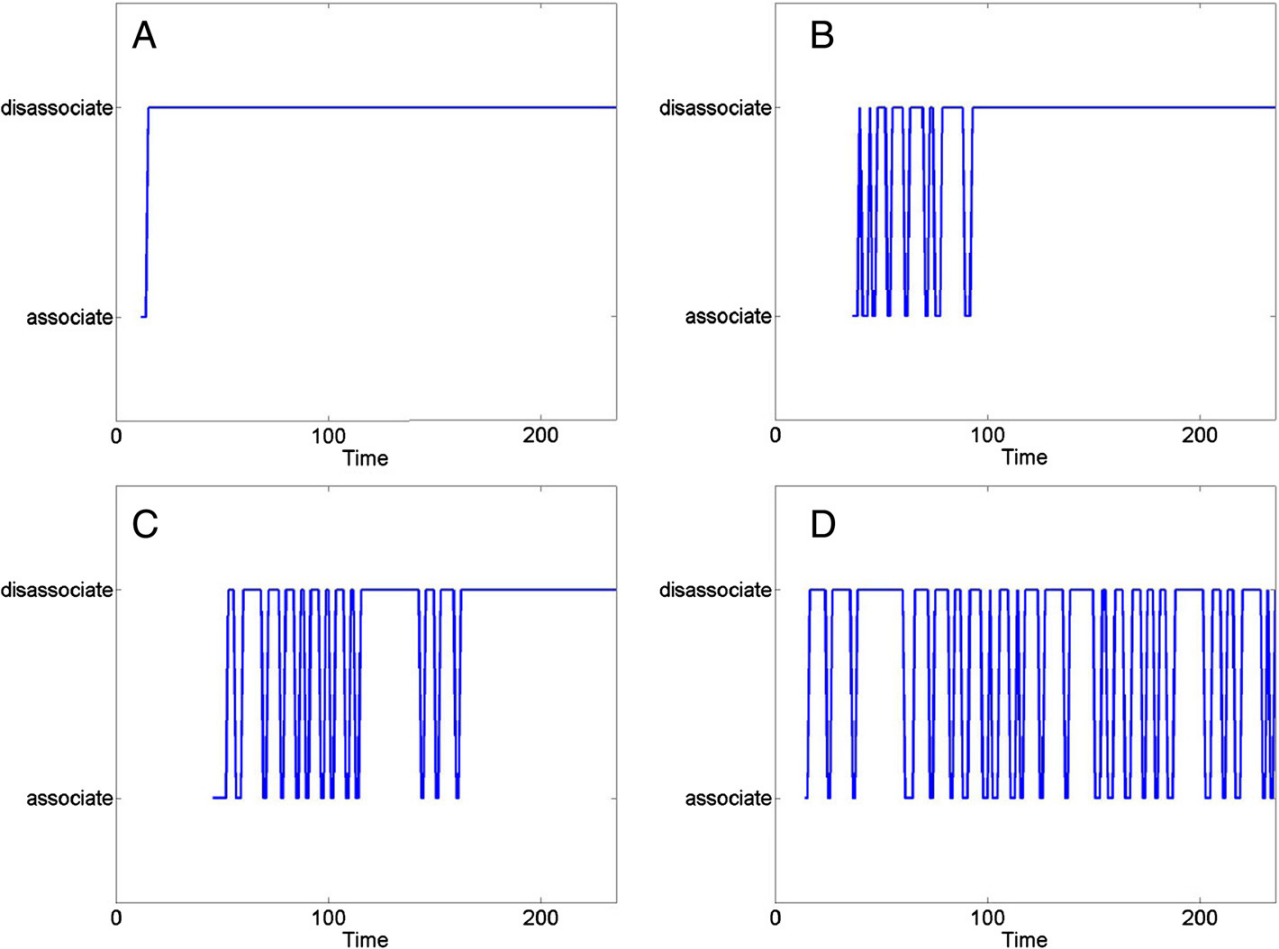
**Emergent oscillatory association-disassociation at the single-neuron level over time (in arbitrary simulation units) (reproduced from [**[46]**]).** This oscillatory behavior is possibly emerges by a conflict in the instructions coded in the genetic regulatory network. Cells response to an instruction to follow the glial fiber in response to factors in their close environment and at the same time to intrinsic signals that direct them to migrate randomly. Each cell interprets the signal differently leading to different periods and frequencies. This phenomena in four representative neurons is shown: Immediate differentiation **(A)**, short delay **(B)**, long delay **(C)** and oscillatory association over the entire simulated period **(D)**.

The model allowed us to seek for a possible explanation to this phenomenon. We hypothesized that this behavior results from a conflict in the instructions the cells receive. The neurons recognize the glial fiber as a guideline for migration and attempt to associate, but at the same time, the reduced activity of the regulator instructs an opposed disassociation (Figure 6). This hypothesis could be tested experimentally by tracking the migration of individual neurons during development, a technique that to my knowledge is not yet feasible. We were unable to find experimental evidence that supports or dispute this finding.

## Conclusions

The research direction reviewed here paves a way for realistic in-silico models for cell population dynamics and organ development. This kind of modeling study, consistent with its early maturity, poses theoretical and technical challenges. Current simulations have provided a qualitative platform to study the development of the organs. In the future, more realistic models should account for quantitative aspects of the development. In particular, the number of cells in the simulations is often a few orders of magnitude less than reality. Increasing the cell population in the simulation introduces technical hurdles related to computational resources. Rapid computational advances could provide the means to tackle this hurdle. Second, the amassed large-scale genomic data (e.g., binding rates, coding regions, DNA sequencing) is collected and organized in many diverse datasets. These databases are often analyzed using vastly different underlying methodologies. Currently, our models overlook large-scale genomic dataset and instead incorporated simplifications when needed. For instance, gene expression is represented as a simplified binary fashion in two states: expressed and unexpressed. Future development of in-silico models should incorporate this genomic data. This can be done, at least at the first instance, by combing classic mathematical tools for rate study such as differential equation models. Lastly**,** our simulations utilized software engineering tools that typically were designed for systematic analysis, design and implementation of computer programs. In the model construction, we have adapted these tools to fit to biological purposes. However, many features of these tools are irrelevant to the modeling process and increase unnecessarily the complexity of the biological simulation. Making these models more accessible to the scientific community will require the development of user-friendly tools to facilitate quick model construction and testing.

Clearly, even when these and other challenges are successfully addressed, the modeling process should be ongoing. These kind of models should be continuously revised, synthesizing newly and theoretical results. The validity of the models will consequently be validated. [13,14,40]. In the long run, these research techniques could benefit medical and educational purposes. The techniques would enable disease modeling and would assist the results of drug trials, thus accelerating effective drug design through shortening development time. In the same vein, it would serve as a platform to familiarize biologists with the emerging fields of in-silico research and computational biology. In a similar manner that flight simulators are broadly used to train pilots, future in-silico models could be used to train experimental biologists, reducing the need for time-consuming and costly laboratory experiments.

## Competing interests

The author declares that he has no competing interests.

## Authors’ contributions

YS has conceived the research direction, designed the models and performed the theoretical experiments and analysis. YS wrote this review.
